# The effect of transport apertures on relay-imaged, sharp-edged laser profiles in photoinjectors and the impact on electron beam properties

**DOI:** 10.1107/S1600577524003904

**Published:** 2024-06-06

**Authors:** Mark Roper, Suzanna Percival, Katherine Morrow

**Affiliations:** a ASTeC, STFC Daresbury Laboratory, Sci-Tech Daresbury, WarringtonWA4 4AD, United Kingdom; b Cockcroft Institute Sci-Tech Daresbury WarringtonWA4 4AD United Kingdom; Paul Scherrer Institut, Switzerland

**Keywords:** electron diffraction, photoinjector, wavefront propagation, electron bunch emittance, Gibbs phenomenon

## Abstract

In a photoinjector electron source, the initial transverse electron bunch properties are determined by the spatial properties of the laser beam on the photocathode. Spatial shaping of the laser is commonly achieved by relay imaging an illuminated circular mask onto the photocathode. However, the Gibbs phenomenon shows that recreating the sharp edge and discontinuity of the cut profile at the mask on the cathode is not possible with an optical relay of finite aperture. Furthermore, the practical injection of the laser into the photoinjector results in the beam passing through small or asymmetrically positioned apertures. This work uses wavefront propagation to show how the transport apertures cause ripple structures to appear in the transverse laser profile even when effectively the full laser power is transmitted. The impact of these structures on the propagated electron bunch has also been studied with electron bunches of high and low charge density. With high charge density, the ripples in the initial charge distribution rapidly wash-out through space charge effects. However, for bunches with low charge density, the ripples can persist through the bunch transport. Although statistical properties of the electron bunch in the cases studied are not greatly affected, there is the potential for the distorted electron bunch to negatively impact machine performance. Therefore, these effects should be considered in the design phase of accelerators using photoinjectors.

## Abstract 2

Wavefront propagation studies show that apertures in the laser injection path into photoinjector electron guns result in spatial ripples in relay-imaged cut-Gaussian profiles. The effect on electron beam properties depends on the extent to which space charge washes out the corresponding ripples in the emitted electron bunch, but could significantly negatively impact the quality of the electron bunch.

## 1. Introduction

There are two methods commonly used for generating electron bunches for electron accelerators: using either photoinjector guns or thermionic guns. Thermionic guns ‘boil’ electrons off a surface through direct application of heat to raise the thermal energy of the electrons above the material work function. Photoinjector guns use a laser to excite the electrons of a photocathode (often a metallic surface) causing electron emission via the photoelectric effect (Clendenin, 1996 ▸). The photon energy of the laser is greater than the binding energy of the electrons or work function of the material. The photoelectric effect can generate higher brightness beams than thermionic processes (Suberlucq, 2004 ▸). This, along with the ability to control properties of the electron bunch through the manipulation of the laser, means photoinjectors are the obvious choice when low emittance electron beams are required. With a homogeneous photocathode, the 3D photoinjector laser distribution at the cathode determines the 3D distribution of electrons generated. Ultrashort laser pulses can be used to generate picosecond and even femtosecond electron bunches at the cathode. The transverse intensity distribution of the laser is imprinted onto the photoemitted electron bunch. Therefore, a high-quality laser beam is needed to generate a high-quality electron bunch.

The ideal distribution for the electron bunch is a 3D ellipsoid (Kapchinskij & Vladimirskij, 1959 ▸), in which the transverse intensity profile is a semi-ellipse. The space charge within a bunch of this shape is linear with position from the beam axis and therefore the emittance of the electron bunch can be preserved. However, shaping the laser to produce such a beam requires a very complex setup. Instead, nearly the same emittance can be achieved by using a cut-Gaussian spatial profile (Ding *et al.*, 2016 ▸). This is because a Gaussian cut at the full width at half-maximum (FWHM) can be readily seen to approximate a semi-ellipse. Cut-Gaussians are much more easily generated by passing a Gaussian laser through a circular mask. In accelerators, the laser pulse often needs to be transported several meters from a laser room into the accelerator bunker and the mask cannot be placed close to the cathode due to the structure of the accelerator and gun cavity. Simply projecting the laser through the mask would result in a diffraction pattern at the cathode. Therefore, to give the highest quality cut-Gaussian laser beam at the cathode, the circular mask is imaged to the cathode plane with an optical relay. As the relay is imaging an object (the circular mask) that can be stably fixed in space, there is the additional benefit of a positionally stable beam on the cathode, free from any pointing jitter of the laser (Hunt *et al.*, 1978 ▸).

**Figure 1:**
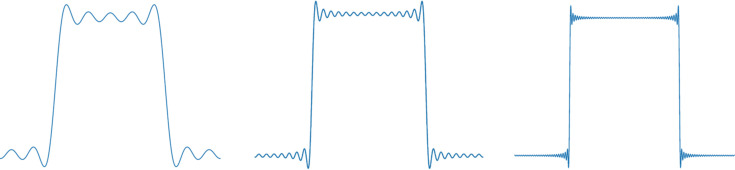
Reconstruction of a square wave using a finite number of harmonic components showing the Gibbs phenomenon. From left to right, reconstruction using: 5 harmonics, 15 harmonics and 50 harmonics.

The sharp edges of the cut laser beam can only be fully reconstructed when an infinite series of spatial Fourier components are present. To illustrate this, Fig. 1 ▸ shows an analogous situation of the reconstruction of a square wave with 5, 15 and 50 harmonic components. A finite Fourier series always leaves anomalies at the discontinuity, manifesting as ripples over the top of the wave and an overshoot at the edges. This effect is called the Gibbs phenomenon (Gottlieb & Shu, 1997 ▸). In the case of the cut laser beam, the spatial harmonic components extend infinitely in space and so, no matter how large the optical components used in the imaging relay, some harmonics will be lost. More significantly, because the laser beam needs to be near normal incidence on the cathode to prevent pulse stretching temporally and spatially, it must pass through small apertures into the accelerator and through the gun, removing more Fourier components. Additionally, the laser beam does not pass through the centre of apertures in the gun cavity, leading to an asymmetric effect on the beam profile. This means the reconstruction of the cut-Gaussian at the cathode will always contain ripples. In this paper we aim to answer the question: what is the real impact of the Gibbs phenomenon on laser beam profiles in photoinjectors and does it negatively impact the quality of the electron beam? We aim to keep this study as general as possible; the intention is to make designers aware that there is a fundamental limit to how well a cut-Gaussian beam can be replicated at the photocathode. However, the study does require some specific parameters to be used and these are based on the design for a proposed Relativistic Ultra-fast Electron Diffraction and Imaging (RUEDI) accelerator (Section 2). The first stage of the study is to model the effect of apertures on the cut-Gaussian beam profile (Section 3). After that, the impact on the transported electron bunch is assessed (Section 4). In Section 5, the results of the study are summarized and the consequences for the design of photoinjector based accelerators discussed.

## 2. The RUEDI electron accelerator

The planned Relativistic Ultra-fast Electron Diffraction and Imaging (RUEDI) facility at Daresbury Laboratory (McKenzie *et al.*, 2023 ▸) is being designed as a combined MeV-level ultra-fast electron diffraction (UED) and microscopy (EM) machine. There are a number of MeV UED facilities operating around the world, for example at SLAC (Weathersby *et al.*, 2015 ▸) and BNL in the USA (Zhu *et al.*, 2015 ▸), DESY in Germany (Manz *et al.*, 2015 ▸), and in Shanghai, China (Fu *et al.*, 2014 ▸) and Daejeon, Korea (Kim *et al.*, 2020 ▸), and the technique is considered complimentary to ultra-fast science performed on free-electron lasers (FELs). RUEDI aims to push the temporal resolution to 10 fs or better. A common aspect of these machines is that they use the same type of photoinjector guns that are employed on FELs to generate the short electron bunches required. The addition of time-resolved MeV EM would be a world-first and open up the possibility of probing dynamics within living cells, which is not possible on conventional EM machines due to the reduced penetration of the lower energy (∼250 keV) electrons.

**Figure 2:**
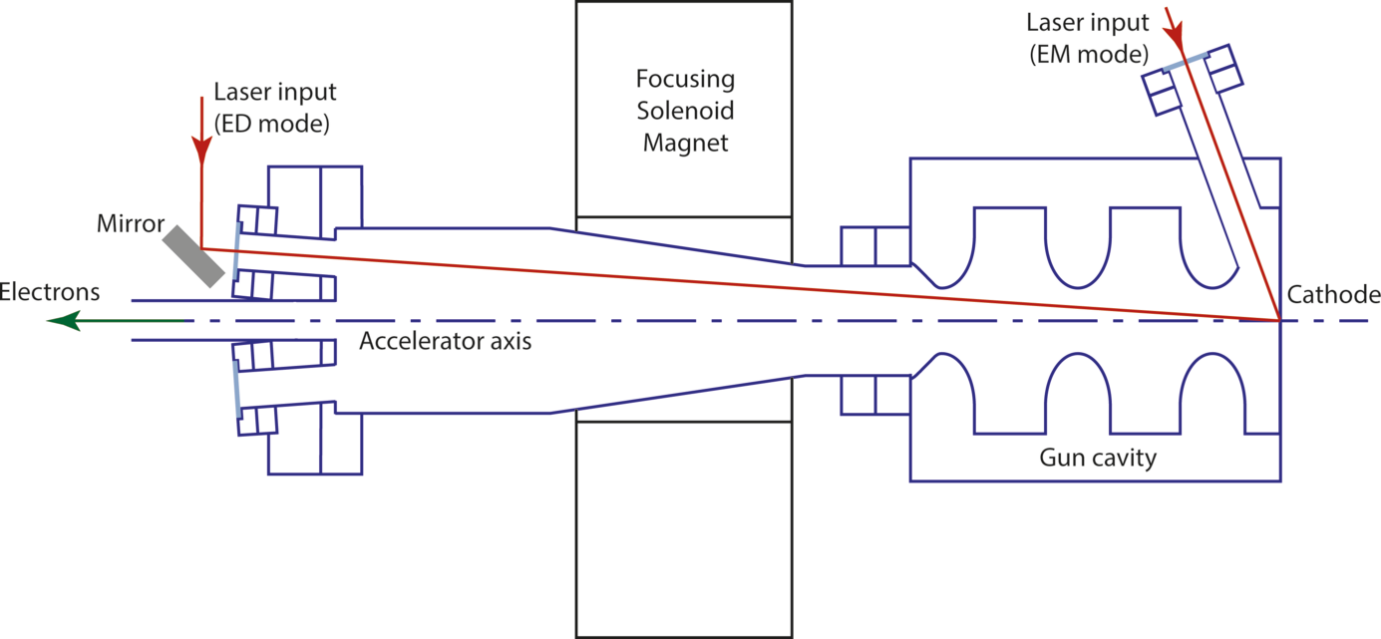
Schema showing the two modes of laser injection into the RUEDI electron gun. Near-normal incidence in ED mode requires the laser to pass through a restrictive port and close to apertures in the gun cavity. Oblique incidence will be used in EM mode as it allows a smaller beam spot on the cathode, but limits the temporal resolution.

RUEDI will be based on an S-band photoinjector gun (Militsyn *et al.*, 2023 ▸) with a copper photocathode from which low emittance (∼10 nm rad) and ultra-short (∼50 fs FWHM) electron bunches are produced and accelerated to 4 MeV for electron diffraction. Electron microscopy (imaging) is performed at 2 MeV and at lower temporal resolution (nanoseconds). A Ti:sapphire photoinjector laser will produce UV pulses at the third-harmonic wavelength 266 nm and with pulse length below 60 fs FWHM. In ED mode, these will be injected into the gun to strike the cathode at near-normal incidence since this avoids the effective pulse stretch from the time of arrival difference across the beam that occurs at oblique incidence. The downside of near-normal injection is that the laser is required to pass through various restrictive apertures to reach the cathode (Fig. 2 ▸). An injection mirror is placed as close as possible to the accelerator axis and this limits the size of the injection port to about 16 mm internal diameter, this being the maximum internal tube bore for a mini-Conflat vacuum flange. Additionally, the RF cavity of the gun has two or three irises (the size and number will depend on the cavity design); the current 2.4-cell cavity design has three irises of about 24 mm diameter. An extra complication is that the beam will not pass centrally through the cavity irises, being closest to the edge of the iris furthest from the cathode. RF couplers can also present a restrictive aperture, but it is planned to use side-coupling on RUEDI.

Achieving the required electron beam properties from the gun for ED mode requires the UV laser to form a cut-Gaussian beam profile on the cathode with the cut at the FWHM and with diameters in the range 0.1 to 0.4 mm. This rather small laser spot means a large aperture will be required to transport sufficient spatial Fourier components to reconstruct the cut-Gaussian profile accurately. Apertures in the injection path are likely to truncate the beam sufficiently for the beam footprint on the cathode to be significantly affected. This could impact the electron diffraction mode of RUEDI where the bunch charge will be very low (femto-Coulomb level), and so space-charge effects might not be sufficient to wash out irregularities in the initial electron bunch. However, the small size of the electron bunch (transversely and longitudinally) could counteract this.

## 3. Modelling the effect of restrictive apertures on the laser beam profile

The impact of restrictive apertures on a transversely coherent optical beam must be studied from a wave-optical perspective and so a wavefront propagation code is required. The code used in this work is the *FOCUS* wavefront propagation code, developed at Daresbury Laboratory (Bowler & Higgins, 2009 ▸). This is a 2D code that uses the full Rayleigh–Sommerfeld equations to propagate the fields rather than numerical approximations for the propagation. The complex field at any surface is represented by its value on a Cartesian grid of points. The Sommerfeld propagation integral (Goodman, 1996 ▸; Born & Wolf, 1999 ▸) is solved to move the field from one surface to the next in the optical system.

A full model of the photoinjector laser relay would require the optical train from illuminated mask to focal plane (cathode) to be modelled with a restrictive aperture added at the appropriate point. This would be time-consuming to run and require an arbitrary assumption about the optical relay (system magnification, overall transport length, focal lengths, position and size of optics). Since this work aims to look at the impact of apertures in a more general way, a simplified model is used to simulate their impact.

First, the required sharp-edged intensity profile at the cathode is generated and becomes the *source* for the propagation. In this study, this means cut-Gaussian beam profiles with widths chosen so that a cut at the FWHM gives profiles of 0.1, 0.2 and 0.4 mm diameter. The wavelength was set to 266 nm, *i.e.* the third-harmonic of a Ti:sapphire laser. These sources are then propagated to various distances to generate the *intermediate* beam, where they are then truncated by apertures of various sizes. The truncation is achieved by zeroing the field components outside the boundary of the aperture. Then the field is reverse propagated back to the original source plane to form the final *image*, which thus shows the effect of the truncation by the aperture.

Although this is a simplified model it is still highly relevant when looking at the impact of apertures in the accelerator itself. Between the point at which the laser is deflected towards the cathode (see Fig. 2 ▸) and the cathode, there can be no optics. Thus, the profile of the laser beam inside the accelerator is determined entirely by the free-space propagation of the profile at the cathode and, vice-versa, the profile at the cathode is determined by the free-space propagation of the profile at any aperture. By starting with the ideal target profile on the cathode, this work shows how this ideal profile is fundamentally compromised by the apertures in the accelerator and can therefore never be achieved in practice.

The model also assumes, in effect, that the relay mask is illuminated by a perfect Gaussian laser beam. UV laser beams used in photoinjectors are generated by harmonic conversion with non-linear optics and this process introduces significant spatial noise in the beam, such as hot-spots. This noise compromises the beam quality at the cathode and should be removed by a spatial filter. Spatial filtering is also highly desirable when longitudinal shaping of the beam is employed and to prevent damage to optics. A spatial filter before the relay mask should always be included in a photoinjector laser transport design if high beam quality at the cathode is a necessity, and so the results of this study are still valid. Residual spatial anomalies after the spatial filter will be of low spatial frequency and will be unaffected by subsequent transport apertures; they will be overlaid on the Gibbs phenomenon ripples.

A final comment on the model is that the ‘source’ (*i.e.* the target image on the cathode) has a flat phase – it is a beam waist. This need not be the case, and in any real relay-imaging system is unlikely to be so. A question that can be asked is whether manipulating the phase in the final image could allow control of the beam profile at a limiting aperture in such a way that the Gibbs phenomenon ripples in the image are changed. However, since the beam size at the cathode is very small compared with the distance to the nearest restrictive aperture, the curvature of the phase required to position the beam waist at the aperture results in an essentially flat phase over the width of the image and hence negligible change to the beam at the aperture location. Applying a stronger curvature to the phase to make the beam small at one aperture will cause the beam to enlarge elsewhere and increase the impact of other apertures.

### 3.1. Effect of symmetric truncation

**Figure 3:**
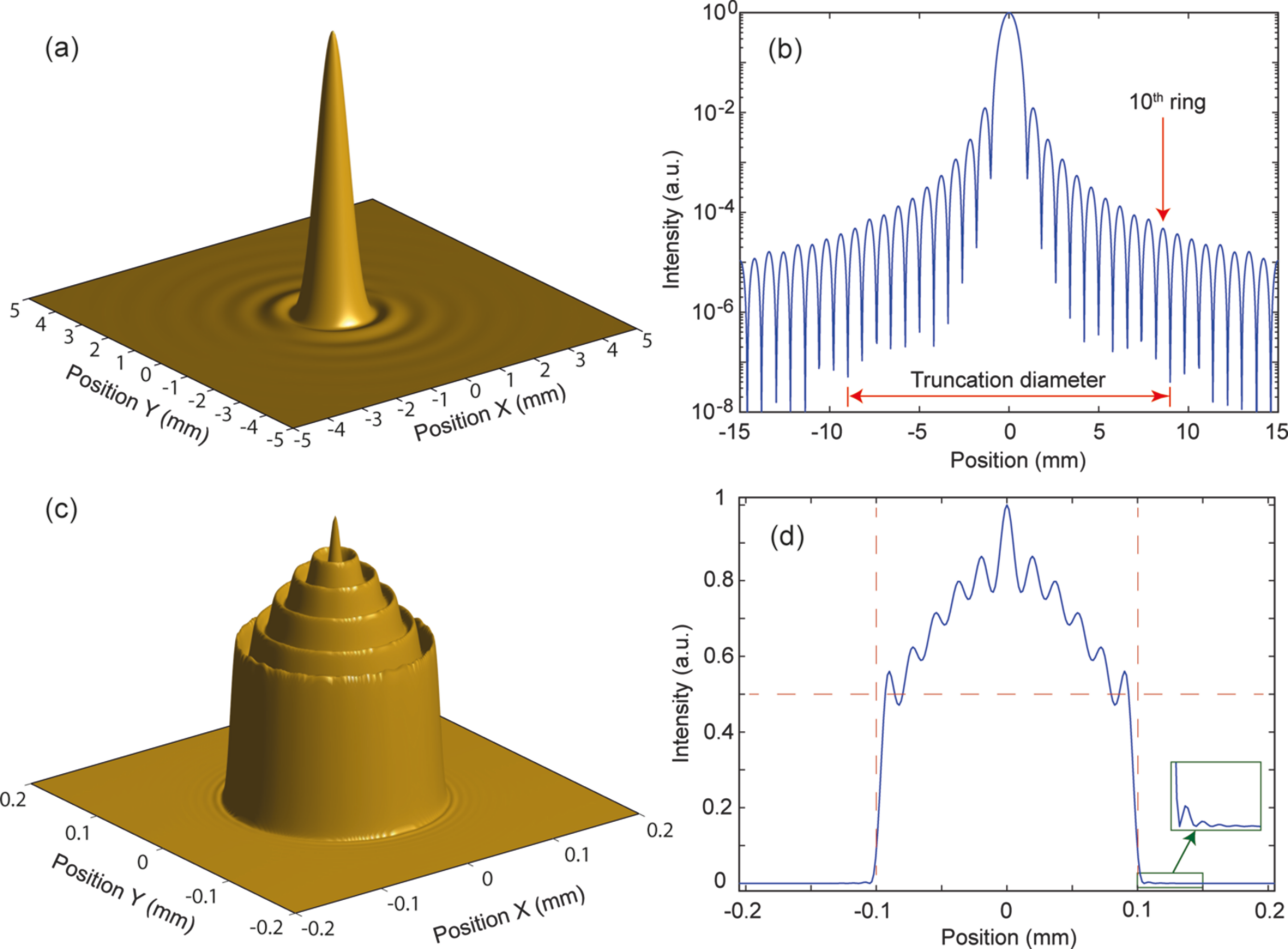
An example of the wavefront propagation results. This is for a 0.2 mm-diameter cut-Gaussian source. (*a*) 3D view of the central 5 mm × 5 mm of the propagation of the source by a distance of 600 mm (intermediate beam). (*b*) Slice through the centre of the intermediate beam to a diameter of 30 mm on a log scale showing multiple rings; an example truncation diameter is shown at the minimum after the tenth ring. (*c*) 3D view of the reverse propagation (image) of the truncated intermediate beam. (*d*) Slice through the centre of the image; the dotted grid lines show the half-maximum and the nominal 0.2 mm target diameter, and the inset shows ripples at the base of the main beam. All intensities are normalized to peak at 1.

The first study looked at the effect of symmetrically positioned apertures, representative of the laser injection port. Fig. 3 ▸ shows an example of this process in which a 0.2 mm-diameter cut-Gaussian source is propagated a distance of 600 mm before truncation and reverse propagation. The intermediate intensity distribution is like an Airy pattern (far-field diffraction of a flat-top profile) with an intense, narrow central peak surrounded by a pattern of weak concentric rings of monotonically decreasing peak intensity. All the intermediate images for propagations into the far-field show this same basic pattern. For the reverse propagation, this pattern is truncated to include only ten rings by a centrally positioned circular aperture cutting at the position of the 11th minima from the centre. Note that the intensity of the peak of the tenth ring is just 0.005% of the main peak, so the truncation is having negligible effect on the transmitted power. However, the image resulting from the reverse propagation now has concentric rings over the peak and a rim around the edge, and there are some weak ripples in the background around the main peak. This is exactly the effect described by the Gibbs phenomenon.

The effects of various truncations of the intermediate beam on the reverse propagated image were explored through three studies: (i) truncating the intensity profile of the intermediate beam at the 11th, 16th and 21st minima from the central peak, so as to include 10, 15 and 20 rings around the central peak; (ii) propagating the source to different distances but truncating the intermediate beam at the same minimum from the central peak; (iii) propagating the different source diameters to the same distance and truncating at the same minimum from the central peak (hence different aperture sizes for each source).

#### 3.1.1. Summary of results from the studies

The reverse propagation (image) always shows common features of concentric rings including a rim around the edge, with either a peak or dip at the exact centre. Furthermore, the number of rings in the image depends exactly on the number of rings of the intermediate beam included within the aperture. Thus, if *N* rings are included within the aperture, the final image has *N*/2 rings about a central peak for *N* even and (*N*/2) + 1 rings about a central dip for *N* odd. Hence, looking at the slices through the peak centre, truncation at the *N*th minima results in *N* local maxima in the slice profile. Referring to Fig. 3 ▸, showing the truncation at the 11th minimum (*i.e.* including ten rings), the image has five rings about a central peak and there are thus 11 peaks in the slice.

Irrespective of the initial propagation distance, the reverse-propagated image is always the same when the far-field intermediate image is truncated at the same minimum from the central peak. For example, truncation to include ten rings in the intermediate image (*i.e.* at the 11th minimum) gives the same final image whether the source was propagated forward and back by 200 mm or 600 mm.

The number of rings in the reverse-propagation is the same for each cut-Gaussian diameter if the intermediate propagation is truncated at the same minimum (to include the same number of rings). Thus, if the intermediate propagations of the 0.1, 0.2 and 0.4 mm cut-Gaussians are truncated at the 11th minimum (ten rings included), the reverse-propagated images all have five rings and are essentially identical apart from the different beam diameters.

### 3.2. Effect of asymmetric truncation

**Figure 4:**
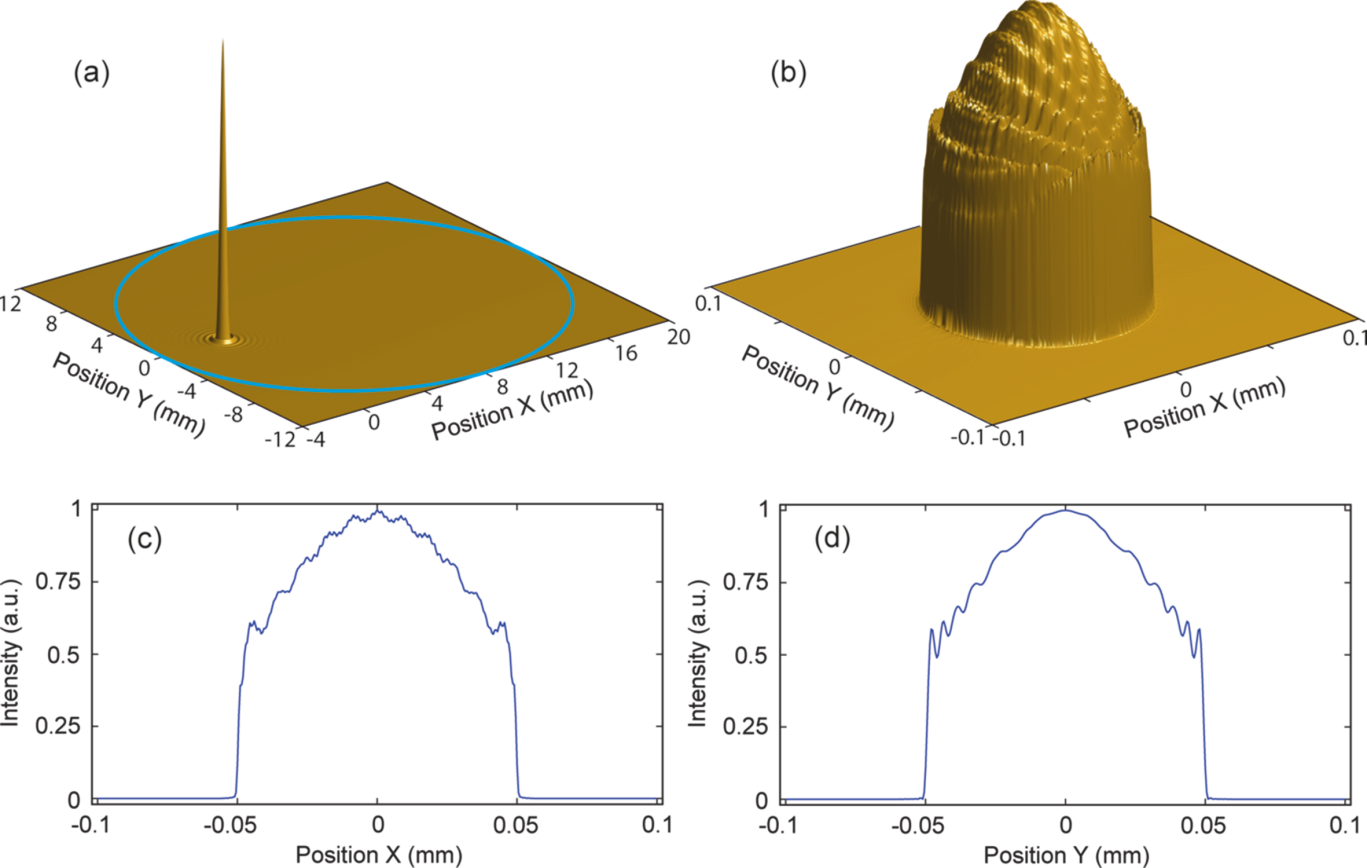
Propagation result for an asymmetrically positioned aperture on the intermediate beam. (*a*) 3D view of the intermediate beam with the offset aperture overlaid, showing that the aperture is very large compared with the core beam size. (*b*) 3D view of the image from the reverse propagation, showing the complex asymmetric ripples. (*c*, *d*) Slices through image centre at *Y* = 0, *X* = 0, respectively.

As already discussed, the restrictive apertures in the laser injection path will not all be symmetric with respect to the laser beam axis. The laser cannot strike the cathode at exactly normal incidence and so must pass closer to one side of the gun cavity than the other. In this case, the final iris in the gun cavity will be the most restrictive aperture and the laser beam may pass quite close to it. Additionally, any axial RF coupler for the cavity is likely to impose a more restrictive aperture (smaller in diameter and further from the cathode). The effect of an asymmetrically positioned aperture will obviously depend on both the aperture diameter and the offset from the laser beam centre. Generalization is therefore not possible. To illustrate the potential consequence, the effect of the final iris in the current 2.4-cell cavity concept for RUEDI is considered. This is 24 mm in diameter and is 125 mm from the cathode. The laser beam strikes the cathode at an angle of incidence of 3.8° and so the laser beam centre is offset by 8.3 mm from the cavity axis at the iris position. Thus the laser beam centre is only 3.7 mm from the edge of the iris. The impact on the 0.1 mm-diameter cut-Gaussian laser beam is shown in Fig. 4 ▸. The asymmetrically positioned aperture produces an asymmetric pattern of ripples over the laser beam, though the modulation is not very strong.

## 4. Electron bunch simulations

Following the results of the *FOCUS* simulations, the next stage was to investigate how these laser intensity distributions affect the generated electron bunch as it propagates. This was done by using the resulting laser distributions as the transverse electron distribution input for the particle tracking code *GPT* (*General Particle Tracer*) developed by the Pulsar Physics group (van der Geer *et al.*, 2005 ▸).

*GPT* contains multiple space charge calculation methods. In this case the three-dimensional particle-in-cell method was implemented which uses a non-equidistant Cartesian mesh around the bunch to calculate the space charge forces in the rest frame of the bunch. These calculations include cathode effects such as mirror charges but are unsuitable for the calculation of granularity/stochastic effects. This method was chosen over the relativistic point-to-point space charge model as a compromise between accuracy and simulation speed. The number of mesh cells in each dimension is calculated by default as the cube root of the number of macroparticles used to represent the bunch. Therefore, the chosen number of macroparticles must give enough mesh cells to accurately depict electron distributions with finer ring patterns. For this reason, it was decided to use 1 × 10^6^ macroparticles in each simulation, which equates to 100 mesh cells in each dimension.

Cylindrically-symmetric laser distributions were transferred from the *FOCUS* simulation results to *GPT* by taking a one-dimensional radial intensity profile from the two-dimensional distribution, such as the one seen in Fig. 3 ▸(*d*), and using this to create a cylindrically symmetric electron bunch. For the asymmetric distributions the intensity variation across the *x–y* plane was represented as a greyscale bitmap image which could then be read into *GPT* directly. In all bunches, the initial particle distribution was created using Hammersley sequences which artificially reduce the shot noise to contend with the statistical noise that arises from each macroparticle representing many electrons. The electron bunch emitted by the cathode is assumed to be identical to the incident laser pulse in terms of longitudinal and transverse intensity distribution and size.

Two different electron bunches were used in the study. The first was based on the bunch used in current RUEDI diffraction mode beam dynamics simulations, with the same bunch dimensions, charge and longitudinal particle distribution, as well as the same fields used for the injector and solenoid. The second bunch had parameters chosen to minimize the charge density of the bunch, and fields chosen to minimize space charge effects. A comparison between the bunch and beamline parameters of the first and second case is given in Table 1 ▸.

In order to study the effect of the rings in the laser beam caused by truncation with symmetrically positioned apertures on the properties of electron bunch, simulations were carried out using laser images generated by reverse propagation of intermediate laser beams with 5, 10, 15 and 20 rings. The images will have either a central peak or dip and a number of concentric rings as described in Section 3.1.1. For comparison, an equivalent bunch was simulated with a smooth Gaussian transverse profile cut at the FWHM point but otherwise the exact same initial parameters. How an electron bunch would be affected by laser profiles truncated with asymmetrically positioned apertures was also simulated for comparison.

### 4.1. Symmetric truncation – RUEDI diffraction bunch

For the first case, the bunch properties and fields acting on the bunch are based on the target properties for the RUEDI diffraction mode beam which are given in Table 1 ▸. This bunch is a very short ‘blow-out’ mode bunch which, due to its short length, is limited to a Gaussian longitudinal profile. When the bunch is emitted, it starts with a very high charge density so that it expands rapidly under its own space charge forces into a 3D ellipsoid, during which the space charge forces of the bunch become linear. This is integral to the beam dynamics of this RUEDI operating mode (Luiten *et al.*, 2004 ▸). The accelerating field comes from the 2.4-cell S-band RF gun discussed in Section 2, followed by a focusing solenoid to counteract the space charge expansion of the bunch, keeping the bunch size approximately constant and not overfocusing.

**Figure 5:**
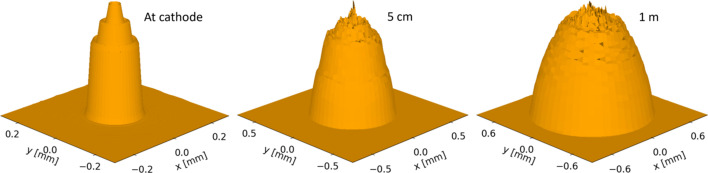
2D intensity profiles of the electron bunch created by a laser with five intensity rings at the cathode and after propagating to 5 cm and 1 m from the cathode. Some noise is introduced to the plots in the process of binning the particles in the bunch to obtain an intensity profile.

The rapid bunch expansion means that any pattern in the particle distribution of the bunch will change and blur as the bunch propagates, as seen in Fig. 5 ▸. The expansion causes the sharp bunch edge and rings to smooth, so that, even for the bunch with five intensity rings, the furthest from a smooth Gaussian simulated in this study, the features are almost entirely blurred out by the time the bunch reaches 1 m from the cathode. A disadvantage of this expansion is an increase in the energy spread and length of the bunch, but this is a feature of using the blow-out mode with or without the laser ripples and the blurring of the ripples is an unrelated benefit.

The only statistical property of the bunch that is noticeably affected by the intensity rings is the RMS projected transverse emittance. Even this is not greatly affected, with only a 1.5 nm rad, or 4.6%, difference between the bunch created using a 15-ring laser distribution, which has the largest emittance at 1 m from the cathode at 34.0 nm rad, and the 20-ring distribution, which has the lowest at 32.5 nm rad. The distributions containing an even number of rings have emittance values closer to the ideal smooth distribution (32.7 nm rad) than the ones with odd numbers of rings, which is likely to do with whether there is a dip or a spike of intensity at the centre of the bunch. The difference in emittance of the 5- and 15-ring distributions and the 10-ring, 20-ring and smooth distributions is not considered statistically significant.

**Figure 6:**
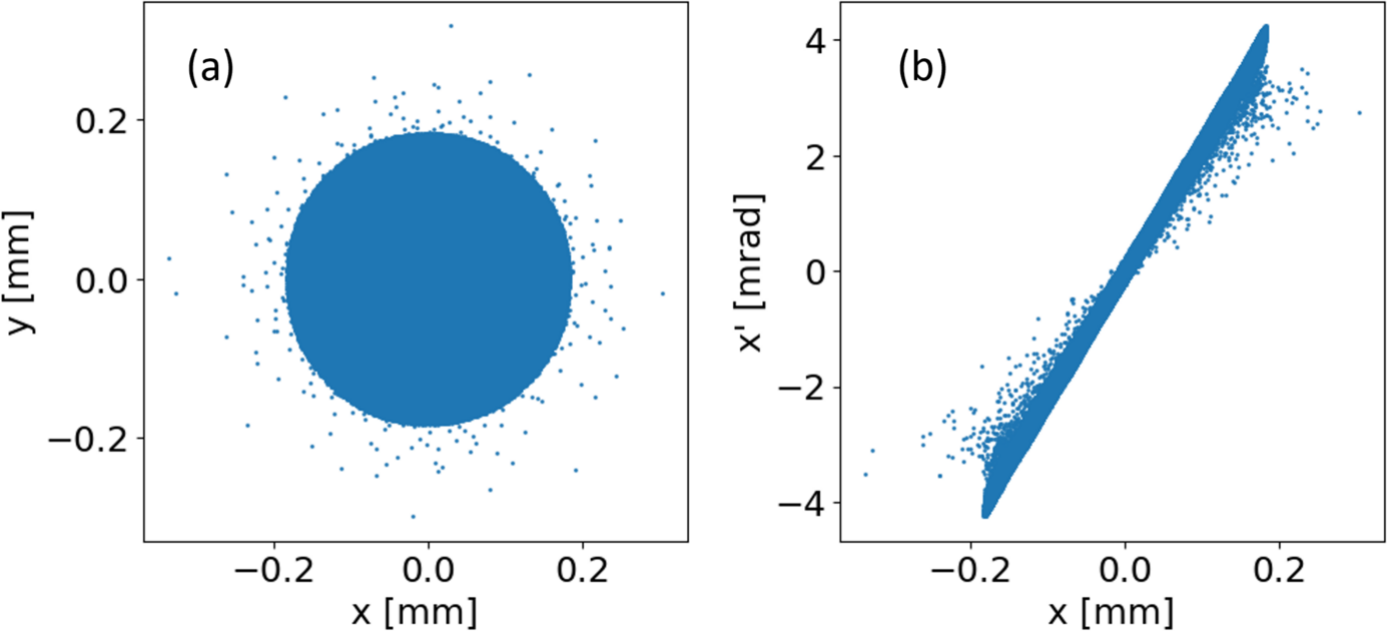
(*a*) Front-view projection and (*b*) transverse phase space of the bunch with the 5-ring ripple structure after being propagated to 1 cm from the cathode.

Owing to the very low laser intensity ripples that exist at radii outside the FWHM cut, as seen in the inset of Fig. 3 ▸(*d*), which are not present in the ideal smooth cut Gaussian profile, additional particles are ‘emitted’ outside the transverse phase space of the particles within the FWHM cut, as can be seen in Fig. 6 ▸. Whilst these start outside of the front-view projection of the main bunch particles, the rapid space charge expansion means that, within 10 cm from the cathode, the rest of the bunch envelopes these stray particles in real space, but they remain outside the transverse phase space of the bunch. It would therefore be very difficult to collimate them from the bunch, but they still contribute large amounts to the RMS projected transverse emittance. There is a high possibility that these extra particles would be comparatively negligible compared with the dark current emitted simultaneously to the beam.

### 4.2. Symmetric truncation – low charge density bunch

The second case looks at an electron bunch which is designed to have a much lower charge density at emission, and so will be acted upon by much weaker space charge forces, whilst keeping parameter values in the realm of those that could be considered for a real machine. This theoretical bunch uses the same transverse intensity profiles as in the first case, stretched to match the new bunch radius, but has a low charge and large dimensions to minimize charge density. A uniform longitudinal profile is used as this makes the space charge forces more linear across the initial bunch than a Gaussian profile. These values, which can be seen in Table 1 ▸, give the bunch a charge density approximately 40000 times lower at emission than the bunch based on the RUEDI diffraction mode. It is therefore expected that this bunch will retain the ring intensity profiles as it propagates for a much longer distance.

To even further decrease the amount of space charge expansion that the bunch will undergo, the 2.4-cell S-band gun used for the first case was changed to a 1.4 cell S-band gun with a much higher accelerating field of 106.4 MV m^−1^. The faster acceleration of the bunch will mean there is less time for space charge forces to cause bunch expansion before the particles become relativistic. The strength of the solenoid field has been adjusted to compensate for these changes, so that the bunch size following the solenoid remains approximately constant. The position and field map used for the solenoid remains the same as used by the simulations in Section 4.1.

**Figure 7:**
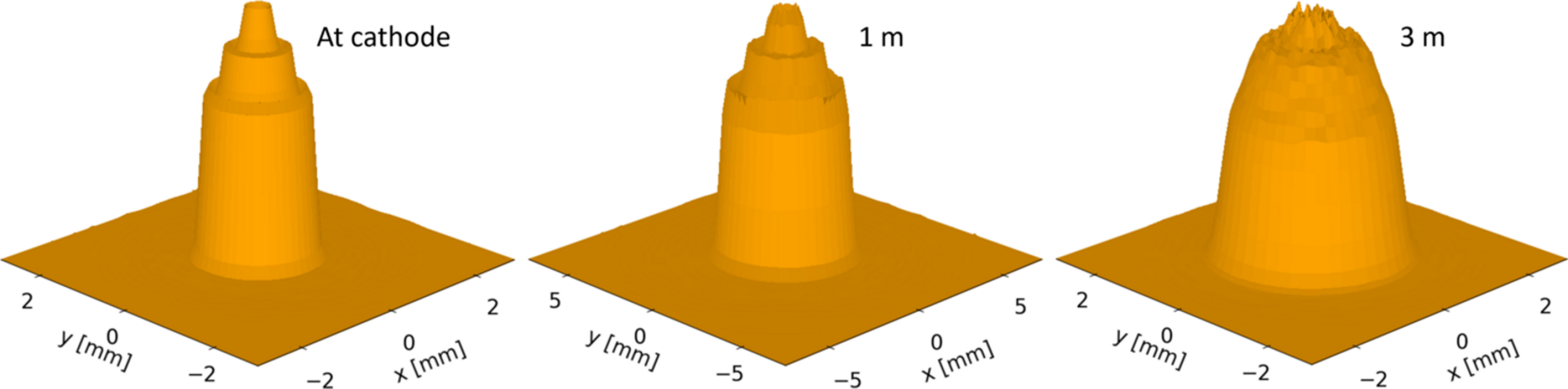
Comparison between the initial 2D particle distribution of the low charge density bunch created using a 5-ring laser profile and the same distribution once the bunch has propagated 1 m and 3 m from the cathode. Some noise is introduced to the plots in the process of binning the particles in the bunch to obtain an intensity profile.

As expected, the initial intensity distribution of the bunch lasted a lot longer for the low charge density bunch, as seen in Fig. 7 ▸. The bunch radially expands slowly enough that there is very little visible change in the rings even when propagated to 1 m, with significant loss of the ring structure only being seen after the bunch has propagated 3 m. This means that, unless the bunch were to pass through beamline elements that would significantly rearrange the transverse distribution of the bunch particles in a way that is not cylindrically symmetric, initial intensity rings will only blur very slowly. As with the first case bunch, the only statistical property significantly affected is the transverse emittance, with this only having a 4.3% difference between the smallest transverse emittance value at 568 nm rad for the smooth cut-Gaussian distribution and the largest 593 nm rad for the 20-ring distribution.

### 4.3. Asymmetric truncation

An example of a laser intensity profile after passing through an asymmetrically positioned aperture can be seen in Fig. 4 ▸. The resulting asymmetric distortions to the beam are a lot smoother than the symmetric rings and so would need the bunch to undergo even less space charge expansion before the intensity rings would become indistinguishable from noise.

Simulations were run using two laser intensity profiles, one as shown in Fig. 4 ▸, and one with the laser beam further offset from the aperture centre so that the beam centre is just 1 mm from the aperture edge, causing much coarser ripples to be formed. All other bunch parameters and beamline elements were based on the low charge density bunch as given in Table 1 ▸. Simulations of both asymmetric distributions showed the ripples to blur out at a similar rate to the symmetric distributions that have similar levels of intensity modulation. Hence the finer pattern of Fig. 4 ▸ blurred at a similar rate to the 20-ring symmetric distribution, being entirely blurred by 2.0 m from the cathode, whilst the evolution of the coarser pattern was closer to that of the 10-ring symmetric distribution. This suggests that the symmetry of the pattern does not matter as much as the modulation amplitude and period.

Another question is whether the asymmetry of the initial bunch causes the properties of the bunch to vary between the *x* and *y* coordinates. The difference between the *x* and *y* RMS emittance values only reaches 0.2% just after bunch emission, and quickly falls to less than 0.02% during propagation, so is negligible. Features of the electron beam transport such as solenoids, which rotate the bunch in the *x–y* plane and so couple the *x* and *y* properties of the bunch, and other magnets such as quadrupoles and dipoles, which have different effects in the *x* and *y* direction, will cause a much larger difference between the *x* and *y* properties of the bunch than this initial difference in the pattern.

## 5. Conclusion and discussion

The Gibbs phenomenon shows that reconstructing a perfectly sharp-edged laser beam profile is not possible in any real optical system. All apertures in the system, including the apertures set by any optics, result in an unavoidable truncation to the Fourier series required to reconstruct a sharp edge and this results in ripple structures in the beam. There is thus a fundamental limit to how well a profile such as a cut-Gaussian can be reconstructed with a relay-imaging optical system. The presence of restrictive apertures in photo-injector laser transports leads to the expectation that the deviation from the target profile will be significant. The wavefront propagation simulations confirm that these effects will occur. The results here are for cases in which the aperture is in the far-field of the cut-Gaussian profile. In this case, and with a symmetrically positioned aperture, the number of rings in the final beam is directly determined by the number of rings in the far-field pattern transmitted by the aperture. This fits in with the idea that the far-field pattern is the Fourier transform of the field in the aperture and so each ring (peak in the slice) represents a spatial wavelength in the field. Truncating to a specific number of rings (peaks) results in a defined number of Fourier components left to reconstruct the final image in the reverse propagation, and hence results in a defined number of rings in the image. Similar effects will occur in the near-field but it is harder to generalize these cases. Similarly, the effect of asymmetric truncation cannot be generalized.

The initial particle density distribution of the electron bunch is determined by the intensity profile of the laser on the cathode and is thus imprinted with the ripples in the laser beam. The extent to which this initial distribution remains the same as the bunch propagates depends on how rapidly the bunch undergoes space charge expansion. This, in turn, depends largely on the initial charge density of the electron bunch, which is thus the critical parameter in determining the impact of the ripples in the laser beam on the final electron bunch properties. For a high enough charge density, any intensity distribution features will quickly blur and so will have no significant consequence on either the statistical properties of the bunch or the smoothness of the particle density in the bunch at, for example, an experimental interaction point. On the other hand, a very low charge density bunch will keep the modulation of the initial intensity distribution for a much longer propagation. So, despite the statistical properties of the bunch also being only slightly altered, there could be an impact on machine performance due to the actual structure of the bunch.

There are additional factors which could affect the space charge expansion, and hence the longevity of the ripples in the bunch, without affecting the initial bunch charge density. For example, the electric field strength of the gun controls the bunch acceleration and faster acceleration reduces the space charge expansion since space charge forces diminish as the bunch energy increases. How much the bunch is focused by the solenoid could also have an effect depending on whether the magnet is used to cancel the radial expansion of the bunch or to overfocus the bunch, since forcing the particles into a waist will cause further blurring due to the strong space charge forces. Additionally, a larger initial energy spread across the bunch would not have a direct effect on the rate at which the ripples blur, but could increase the blurring through chromatic solenoid lens aberrations. However, since the extent to which initial structures in the bunch will be transported along the accelerator will depend on the details of the magnetic lattice, specific simulations should be performed to assess the actual impact.

Given that the effects studied here show that relay-imaging an illuminated mask onto the cathode can never give a perfect beam profile on the cathode, the question to ask is whether such an approach is a good one. In fact, relay imaging an illuminated mask has some very clear advantages. Firstly, it is a very simple way of achieving (approximately) the required laser beam profile, requiring no complicated optics. It is thus also reliable and consistent, both of which are important factors when operating accelerators. Secondly, it has the major advantage of stabilizing the beam position on the cathode since the mask defines the beam outline and can be fixed in space relative to the cathode with very high precision. A highly stable laser beam position at the cathode is the first requirement for a stable electron bunch from the gun.

Additionally, it should be noted that the effects modelled here are for the case of a perfectly sharp truncation of the intermediate field, this being an idealized representation of the beam mask. A real mask will not have such a perfect cut-off of the laser beam due to edge roughness and its finite thickness and this could modify the rings seen in the wavefront propagations. Furthermore, there are many apertures in a real laser transport (including any mirrors, lenses and windows), all of which will affect the final image in a different way. Thus, we can reasonably postulate that the total effect on the final image will be some mixture of multiple different effects that will act to give a more complex but weaker total modulation. Certainly, our practical experience with the photoinjector laser on the CLARA accelerator at Daresbury Laboratory shows that the types of effects predicted here are visible in the beam on the cathode but tend to be less dramatic.

Nevertheless, the consequence of the Gibbs phenomenon when relay imaging an illuminated mask should not be ignored. The performance of the accelerator is ultimately limited by the quality of the photo-emitted electron bunch which is in turn determined by the spatial and temporal profiles of the laser beam. Therefore, a study like this, including both the wavefront modelling of the laser image and the electron bunch transport simulations to produce particle density distributions, will be important during the design phase of any high-performance accelerator to assess the potential impact on the electron bunch, even if it likely to be a worst-case scenario.

As a practical point, it is normal to have a ‘virtual cathode’ in the photoinjector laser transport system in the form of a scintillator screen positioned after a beam splitter such that it is also at the focal plane of the optical relay. It is assumed that the laser beam at the virtual cathode is an exact copy of the beam on the actual cathode and can thus be used to measure the laser beam position and profile. This is important for photoinjector laser system operation since it is impossible to directly measure the UV laser on the cathode itself. The virtual cathode image thus provides the only measure of the laser beam quality at the cathode during machine operation. This work shows that, for the profile at the virtual cathode to be a true copy of the laser footprint on the cathode, the laser path to the virtual cathode should replicate the apertures in the path to the actual cathode as accurately as possible.

## Figures and Tables

**Table 1:** Parameters of the electron bunch, accelerating RF field and solenoid field for the two cases simulated Case 1 uses bunch parameters used in RUEDI diffraction mode simulations and Case 2 uses parameters designed to minimize the space charge expansion of the bunch.

	Case 1	Case 2
	RUEDI diffraction mode	Low charge density
Charge (fC)	400	400
Energy at gun exit (MeV)	4	4
FWHM laser spot size (mm)	0.1	1
Laser pulse length (ps)	0.025 (RMS)	10 (FWHM)
Longitudinal distribution	Gaussian	Uniform
RF gun phase	+22°	+0°
Maximum RF field on axis (MV m ^−1^)	69.07	106.4
Solenoid field strength (mT)	188	277
